# Laparoscopic Appendicectomy Using Endo-Ring Applicator and Fallope Rings

**DOI:** 10.4103/1319-3767.45053

**Published:** 2009-01

**Authors:** Iyoob V. Ali, Joji I. Maliekkal

**Affiliations:** Department of General Surgery, Medical College, Calicut, Kerala, India

**Keywords:** Appendicectomy, endo-ring applicator, fallope rings, laparoscopy

## Abstract

**Background/Aim:**

Wider adoption of laparoscopic appendicectomy (LA) is limited by problems in securing the appendiceal base as well as the cost and the duration compared with the open procedure. The objective of this study was to assess the feasibility and efficacy of a new method for securing the appendiceal base in LA, so as to make the entire procedure simpler and cheaper, and hence, more popular.

**Methods:**

Twenty-five patients who were candidates for appendicectomy (emergency as well as elective) and willing for the laparoscopic procedure were selected for this study. Ports used were 10 mm at the umbilicus, 5 mm at the lower right iliac fossa, and 10 mm at the left iliac fossa. Extremely friable, ruptured, or turgid organs of diameters larger than 8 mm were excluded from the study. The mesoappendix was divided close to the appendix by diathermy. Fallope rings were applied to the appendiceal base using a special ring applicator, and the appendix was divided and extracted through the lumen of the applicator.

**Results:**

The procedure was successful in 23 (92%) cases, and the mean duration of the procedure was 20 minutes (15–32 minutes). There were no procedural complications seen during a median follow-up of two weeks. The equipment and rings were cheaper when compared with that of the standard methods of securing the base of the appendix.

**Conclusion:**

LA using fallope rings is a safe, simple, easy-to-learn, and economically viable method.

Although laparoscopy is increasingly being used for appendicectomy[1–4] by some surgeons, it has not been accepted by many others because of the concerns in safe securing of the appendiceal base, cost-effectiveness, stump complications, and the duration of the procedure. Dealing with the base of the appendix is a crucial step in the entire procedure for which various methods such as Endo-Staplers,[5] Ligasure,[6] Endo-loops,[7] Harmonic Scalpel,[8] etc. are employed. Some of these methods are costly and others are cumbersome. Our aim was to assess the feasibility of a new technique in terms of simplicity of securing the base of the appendix, cost, and efficacy so as to make the procedure more popular and surgeon-friendly.

## PATIENTS AND METHODS

**Figure 1:**
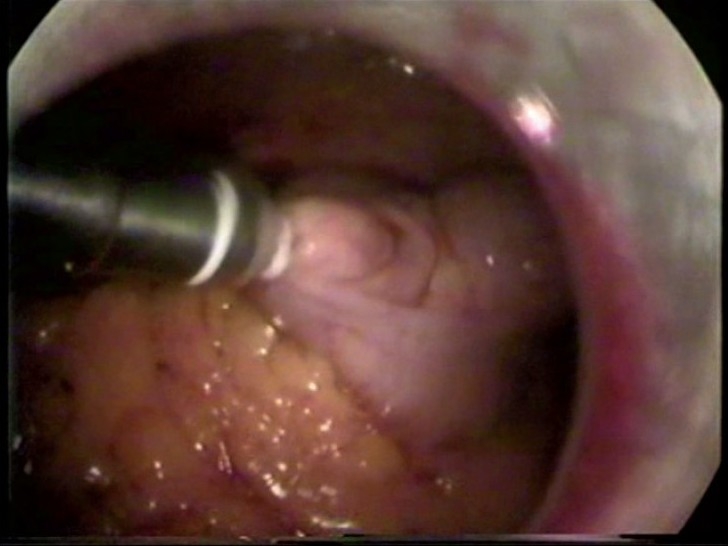
Silicon rings are being applied to the base of appendix using endo-ring applicator

This prospective study was conducted on 25 patients undergoing appendicectomy from January 2006 to April 2007 in a single surgical unit. Approval of the institutional ethical committee was obtained for the study. Both emergency as well as elective procedures were included in the study. A 10-mm umbilical port was introduced by using the open method under general anesthesia and CO_2_ gas insufflated to a pressure of 12–14 mm Hg. Initial visualization of the abdominal viscera was done by using a 10-mm scope. Patients were put in Trendelenberg and right side up position. Two more ports were introduced under vision: one 10-mm port at the lateral border of the left rectus below the umbilicus and another 5-mm port in the lower part of the right iliac fossa. If a highly friable and perforated appendix or a turgid appendix more than 8 mm in diameter was found during the exploration (size compared to the tip of the instrument), they were managed by using the endo-loop or open procedure. The mesoappendix was divided close to the appendix with diathermy. After complete skeletonization of the appendix, an endo-ring applicator (ERA) loaded with two sterile fallope rings was introduced through a 10-mm port on the left side, and the appendix was grasped through the lumen of the ERA with Allis forceps. Two sterile fallope rings were applied close to the base of the appendix, 5 mm apart, and the appendix was divided between the rings [Figure 1]. The appendix was withdrawn into the lumen of the ERA and removed from the peritoneal cavity, avoiding port site contamination. Irrigation and suction were done at the end. Single or three doses of antibiotics were given according to the hospital's protocol. Patients were discharged on the second or third postoperative day. All patients were followed up after two weeks by another surgeon who was blinded regarding the specific method.

## RESULTS

Of the 25 patients in the study, 14 (56%) were men and 11 (44%) were women; the mean age was 24.4 years. Thirteen (52%) patients were for emergency and 12 (48%) for the elective surgery. Of the 13 acute cases, 10 cases were with early symptoms and three cases were on medical treatment for 2–3 days. In two of the acute cases (8%), the appendix was found to be friable and perforated on exploration. Of these two acute cases, one case was dealt with by using an endo-loop, and the other case was converted to an open procedure. Eleven patients out of the 13 acute cases showed early inflammatory changes. Elective cases showed no gross abnormality on exploration. The procedure completed by using the endo-ring method in 23 (92%) patients; a single surgeon performed all the procedures. The mean duration of the procedure was 20 (15–32) minutes. There were no intraoperative complications, and the mean hospital stay was 2.1 days; there were no readmissions. A review of the pathology reports showed 15 cases of acute appendicitis and 10 cases of chronic appendicitis. On follow-up after two weeks, two patients were found to have inflammatory reactions at the umbilical port sites.

## DISCUSSION

**Figure 2:**
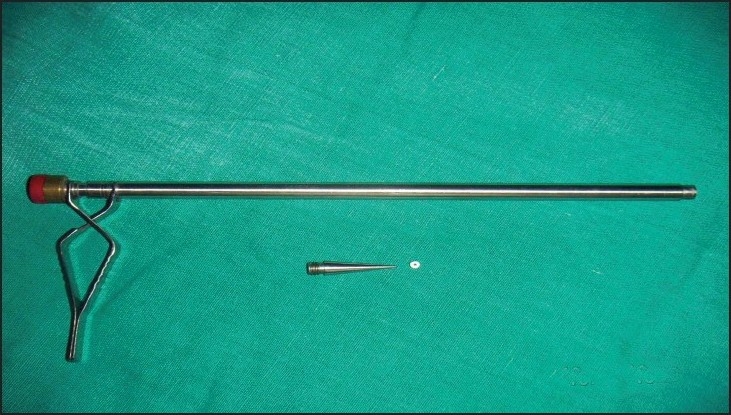
Endo-ring applicator with fallope ring

The ERA is a simple, modified, pile-banding instrument made of stainless steel [Figure 2] whose inner diameter is 8 mm and outer diameter is 10 mm. It can be fully dismantled, washed, and autoclaved. Two rings can be loaded at the same time to the tip of the instrument. The cost of the applicator is around Rs. 5000. Fallope Rings (G. Surgiwear Limited, India) are biologically inert and have been used for laparoscopic tubal sterilization for many years; they cost Rs. 50 per pair. Rings can be precisely applied by ERA so that the remnant is short and hence aids in avoiding late stump complications. As the ring is tight and secure, only one ring is enough for the appendiceal base, unlike the requirement for two endo-loops. In this new method, as the appendix is withdrawn through the ring applicator that is inside the 10-mm port, wound contamination is totally avoided. If a 5-mm telescope is used instead of a 10-mm one, one 10-mm port can be avoided, thus minimizing the wound size.

On comparing with other methods, stump complications following endo-loop appendicectomy have been reported because of the long residual stump following two loop applications[5] and incomplete closure of stump by the knot.[9] Endo-loops with catgut, Vicryl, PDS, etc. are the more commonly used loops. Commercially available preparations are costly, and handmade loops with catgut are commonly used in India. Two loops to the stump and one to the distal portion are often applied because of the concern for the safety of the knot.[9] Correct positioning of the loop to the base of appendix is difficult, and as the deployment of the loops is more time-consuming than ring application, the total duration is more –78.5 vs 20 minutes [Table 1], which influences the final cost. Thus, the main advantages of endorsing the Endo-loop procedure are the ease of the single application, shorter duration, and precise positioning, thus reducing the stump length.

**Table 1:** Comparison of duration of laparoscopic appendicectomy using various methods

Authors	Year	Method	Duration (min)
Karim *et al*.[4]	1996	Loop	78.5
Kurtz TM *et al*.[1]	2001	Staplers	58 ± 4
Shalaby *et al*.[7]	2001	Endo GIA	24 ± 3
		Endo-loop	52 ± 5
Yang *et al*.[6]	2005	Ligasure	47 (22–120)
Present study	2007	Endo-ring	20 (15–32)

Endo-Stapler are now widely used for LA in developed countries. Although the procedure is easy to perform, they cost about €306 per pack and need a 12-mm port for introduction.[7,9] Complications with Endo-Stapler have also been reported.[9] In recent years, Ligasure and harmonic scalpel have bee used to secure the base of the appendix and mesoappendix. The use of these equipments is limited by the high cost of installation.

## CONCLUSION

Laparoscopic appendicectomy using ERA and Fallope rings is a safe and efficient method. This procedure is simple, less time-consuming, and cost-effective. As the technique is similar to the banding of hemorrhoids, it can be expected to have a shorter learning curve. It is probably the cheapest of all the other techniques described so far. The only limitation of the procedure encountered so far is that it cannot be employed for friable and turgid appendices more than 8 mm in diameter.
